# Secondary Genome-Wide Association Study Using Novel Analytical Strategies Disentangle Genetic Components of Cleft Lip and/or Cleft Palate in 1q32.2

**DOI:** 10.3390/genes11111280

**Published:** 2020-10-29

**Authors:** Yunju Yang, Akiko Suzuki, Junichi Iwata, Goo Jun

**Affiliations:** 1 Department of Epidemiology, University of Texas Health Science Center at Houston, Houston, TX 77030, USA; Yunju.Yang@uth.tmc.edu; 2 School of Dentistry, University of Texas Health Science Center at Houston, Houston, TX 77054, USA; akikosuz925@gmail.com (A.S.); Junichi.Iwata@uth.tmc.edu (J.I.)

**Keywords:** orofacial cleft, mixed model genome-wide association study, expression imputation, transcriptome-wide association study, PrediXcan, EPACTS

## Abstract

Orofacial cleft (OFC) is one of the most prevalent birth defects, leading to substantial and long-term burdens in a newborn’s quality of life. Although studies revealed several genetic variants associated with the birth defect, novel approaches may provide additional clues about its etiology. Using the Center for Craniofacial and Dental Genetics project data (*n* = 10,542), we performed linear mixed-model analyses to study the genetic compositions of OFC and investigated the dependence among identified loci using conditional analyses. To identify genes associated with OFC, we conducted a transcriptome-wide association study (TWAS) based on predicted expression levels. In addition to confirming the previous findings at four loci, 1q32.2, 8q24, 2p24.2 and 17p13.1, we untwined two independent loci at 1q32.2, *TRAF3IP3* and *IRF6*. The sentinel SNP in *TRAF3IP3* (rs2235370, *p*-value = 5.15 × 10^−9^) was independent of the sentinel SNP at *IRF6* (rs2235373, r^2^ < 0.3). We found that the *IRF6* effect became nonsignificant once the 8q24 effect was conditioned, while the *TRAF3IP3* effect remained significant. Furthermore, we identified nine genes associated with OFC in TWAS, implicating a glutathione synthesis and drug detoxification pathway. We identified some meaningful additions to the OFC etiology using novel statistical methods in the existing data.

## 1. Introduction

Orofacial cleft (OFC) is a group of congenital deformities showing a gap or breakage in oral features. The gap most commonly appears in the palate (CP; cleft palate), which is the “roof” of the mouth, upper lip (CL; cleft lip) or both (CLP; cleft lip and cleft palate). The majority (~70%) of OFC is nonsyndromic, without additional features [1]. Although it varies among populations, the prevalence of all OFC makes it one of the most common birth defects (approximately 1.2 out of 1000 live births worldwide) [2]. OFC is a significant public health challenge since newborns with these anomalies encounter substantial surgical, nutritional, dental, speech, medical and behavioral challenges in the later phases of their lifetimes, leading to nominal economic and societal burdens [3]. The formation of the head and the face in an embryo is a biological event elaborately coordinated by signaling and pathways [1]. The etiology of OFC is considered a complex disorder that involves not only genetic influences but also interactions with environmental factors.

Studies have shown that genetic components play a significant role in OFC; however, the specific genetic architecture behind the etiology is still unclear. OFC showed a strong familial aggregation in that relatives of cleft cases showed higher risks of being affected than the general population, and the risk decreased as the genetic distance increased [4,5]. Twin studies also confirmed the role of genetics in the OFC status in monozygotic twins that showed higher concordance rates than dizygotic twins [6]. While segregation analyses rarely found definitive results [7], linkage studies mapped several loci for OFC [8,9,10]. Recently, genome-wide association studies (GWAS) in OFC have successfully identified common and rare variants, and their interactions with environmental factors [11,12,13,14,15,16,17,18,19,20,21,22,23,24,25,26]. Besides, there have been active investigations on candidate genes such as *RUNX2, GREM1, MTHFR,* folate-related genes, *ESRRG* and *SNAI1* [27,28,29,30,31]. However, the functional implications of these loci are still obscure, except for *IRF6* [32,33]. In addition to association studies with human genotypes and phenotypes, the functional mechanisms of OFC genes were investigated using animal models. Earlier animal studies have demonstrated the role of *Irf6* gene in epithelial proliferation and differentiation, which are crucial for craniofacial morphogenesis and ectodermal formation [34]. An understanding of the *Irf6* mechanism has been furthered by a recent finding that showed the role of *Irf6* in the osteoblast differentiation of craniofacial bones [35]. Although animal models can help us understand the functional mechanisms involved in human OFC etiology, there are certainly limitations such as the limited number of homologs of human OFC genes and the inability to evaluate the roles of common human genetic variations in animal models.

We performed secondary analyses on Center for Craniofacial and Dental Genetics (CCDG) data on dbGaP (accession: phs000774.v2.p1) in order to understand the contributions of different genetic components to OFC using novel statistical strategies. Our study is aimed at improving the current understanding in OFC genetics for the following reasons. First, we performed a mega-analysis of samples from multiple ethnic groups together by an applying mixed model-based genetic association analysis with an estimated genetic relatedness matrix. The previous study [19] analyzed case-parent trios and the unrelated case-control set separately, and then meta-analyzed them. Furthermore, to adjust for the population structure in the case-control analysis, the principal components (PCs) of ancestry were estimated using the maximal subset of unrelated subjects and a pruned set of SNPs. Our study design reduces the complexity of the analysis while taking the full set of genetic information. Second, we performed a conditional analysis to examine independent genetic influences among multiple OFC loci. Last, we explored the functional implications of human genetic variations on OFC etiology utilizing a computational model that predicted gene expression levels based on the genetic variation in various types of human tissues.

## 2. Materials and Methods

### 2.1. Data Acquisition and Subjects

Genotype and phenotype data were accessed from the dbGaP repository with the accession ID phs000774.v2.p1. The repository includes nonsyndromic cleft cases and their noncleft relatives, as well as controls (*n* = 11,925), mostly from studies led by the University of Pittsburgh Center for Craniofacial and Dental Genetics (CCDG) and also from genetic studies by the University of Iowa. The detailed specifics of the cohort were previously described [19,36], but to summarize briefly, samples were genotyped for 589,945 SNPs on the Illumina Human610-Quadv.1_BBeadChip (Illumina Inc., San Diego, CA, USA), and genotypes were phased using SHAPEIT (Olivier Delaneau, Paris, France and Oxford, United Kingdom). Phased genotypes were imputed with IMPUTE2 software (Bryan Howie and Jonathan Marchini, Oxford, UK) using the 1000 Genomes Phase 1 reference panel (The 1000 Genomes Project Consortium). QC procedures of genotype data are publicly available online at the CCDG study website (http://www.ccdg.pitt.edu/docs/Marazita_ofc_QC_report_feb2015.pdf). The types of nonsyndromic OFC were originally categorized as either cleft lip, cleft palate, or cleft lip and palate. In this study, any cleft types (cleft lip and/or palate) were treated as a case (“OFC”), and subjects without any OFC phenotype as a “control”. Subjects with both the genotypes and phenotypes (*n* = 10,542) were included in this study.

### 2.2. Conditional Genome-Wide Association Study Based on Efficient Mixed-Model Association Expedited (EMMAX) Model

We used the Efficient Mixed Model Association eXpedited (EMMAX) algorithm for the mixed model analysis of common variants (minor allele frequency > 0.01) [37]. The population structure and sample relatedness were modeled using a kinship matrix estimated with the Balding–Nichols algorithm, which is the default method of the ‘make-kin’ function in the EPACTS pipeline that includes the EMMAX software (Hyun Min Kang, Ann Arbor, MI, USA) (https://genome.sph.umich.edu/wiki/EPACTS). Variants with minor allele frequencies greater than 0.01 and call rates greater than 95% were used to construct the kinship matrix. Modeling this genetic covariance as a random effect, the fixed SNP effect is tested for association with the binary cleft trait. The EPACTS pipeline includes a suite of association tests for genetic association analyses for population-level samples including EMMAX. An association p-value smaller than 5.0 × 10^−8^ was considered as genome-wide significant. To investigate the dependence among risk loci, we also performed conditional GWAS, adjusting for the effect of the sentinel association. This process was repeated for nine rounds in order to identify ten independent loci.

### 2.3. Transcriptome-Wide Association Study (TWAS) Using Predicted Expression Levels

We estimated gene expression levels from our genotype data based on the publicly available tissue-specific gene expression database (i.e., GTEx version 6 data) (Broad Institute, Boston, MA, USA), and we performed a transcriptome-wide association study of OFC using PrediXcan (Eric R Gamazon, Chicago, IL, USA) [38,39]. We chose four tissue types (i.e., whole blood, skeletal muscle, visceral adiposity and subcutaneous adiposity) that were relatively more relevant to OFC. Since the current gene expression prediction model cannot model sample relatedness and most of the GTEx samples were from Caucasians [38], we applied PrediXcan analyses on unrelated Caucasian CCDG samples only (*n* = 1220). To adjust for the cryptic population structure in this population, 10 principal components (PCs) were calculated and adjusted for in the following association analysis. Association tests were performed using the ‘glm’ function of the software R (Ross Ihaka and Robert Gentleman, Auckland, New Zealand). The number of genes included in four tissue-specific TWASs were 6736 in whole blood, 7220 in subcutaneous adipose tissue, 4527 in visceral adipose tissue and 6611 in skeletal muscle tissue. The significance thresholds for TWASs were set at Bonferroni levels with the number of genes in each tissue-specific transcriptome (*p*-value < 7.42 × 10^−6^ for a whole-blood TWAS, 6.93 × 10^−6^ for a subcutaneous adipose tissue TWAS, 1.10 × 10^−5^ for a visceral adipose tissue TWAS and 7.56 × 10^−6^ for a skeletal-muscle tissue TWAS). Significant genes were tested for enrichment based on the gene ontology data (MSigDB c5) using the GENE2FUNC function of FUMA GWAS [40,41]. For the candidate gene analysis, we used all genes reported from previous linkage and association studies (Table S1). We searched the 27 overlapping candidate genes from our PrediXcan analysis results. The thresholds used in the candidate gene analyses were adjusted with a Bonferroni correction based on the number of candidate genes in each tissue data, so significance thresholds were 0.005 for skeletal muscle and 0.003 for the remaining three tissues.

## 3. Results

We included 10,542 subjects in the mixed model association analysis, and the demographic characteristics are described in Table 1. The male-to-female ratio is similarly balanced in both the case and control groups (43.3% and 58.5%, respectively). The mean ages of the case and the control groups are 12.06 and 28.66 years (standard deviations of 13.03 and 16.95 years), and the control group is significantly older because it includes parents without cleft. This study population contains nine different ethnic groups: Caucasians and Asians largely represent the population (43.93% and 19.13%, respectively), while Africans, Native Americans and mixed populations compose the remaining 36.94%.

**Figure 1:**
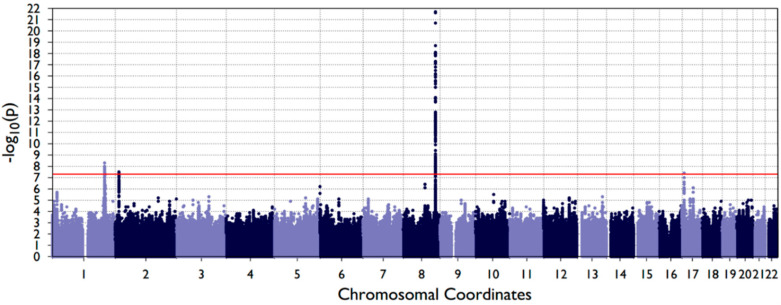
Manhattan plot of OFC GWAS using the EMMAX model. SNPs are positioned along the *x*-axis according to chromosomal position, with −log10 (*p*-value) along the *y*-axis. The genome-wide significance threshold (*p*-value = 5 × 10^−8^) is presented as a red horizontal line.

We identified 289 genome-wide significant associations for OFC (Figure 1) at previously reported loci [19]. The population structure was adequately controlled, and no systematic inflation was observed in the Q-Q plot (Figure S1). The majority of significant associations, 240 out of 289, were from the genomic region called the “gene desert” (8q24) [42]. Other genome-wide significant associations were mapped to *IRF6*, *TRAF3IP3*, *FAM49A* and *NTN1* (Table 2). The sentinel SNPs in *TRAF3IP3* (rs2235370, *p*-value = 5.15 × 10^−9^) were reported collectively as *IRF6* in the previous GWAS [19]. The sentinel SNP for *TRAF3IP3* (rs2235370) was not in LD with the sentinel SNP for *IRF6* (rs2235373) in any population (r^2^ = 0.07 in European, 0.31 in Asian, 0.05 in African and 0.24 in Hispanic), indicating that the two association signals were independent. At a less stringent significance threshold (*p*-value < 1.00 × 10^−5^), 21 association signals, including loci in *PAX7* and *DCAF4L2*, were identified.

To further understand the individual genetic contributions of these markers, we performed conditional analyses. In Table 3, the top ten associations from each sequential conditional GWAS are presented. Manhattan plots from individual conditional analyses are shown in Figure S2. The first genome-wide analysis conditioned on the most significant marker (rs72728755 at 8q24, *p*-value = 2.70 × 10^−22^) identified the next independently significant association, rs7552, which was in the 3′-UTR of *FAM49A*, a protein-coding gene at 2p24.2 (*p*-value = 2.67 × 10^−8^). This signal was also genome-wide significant but was the fifth significant signal in the first analysis. This may indicate that 8q24 biases the effect of 2p24.2 toward the null association when tested together. Conditioning on the first two variants, rs72728755 and rs7552 (8q24 and 2p24.2), the next most significantly associated variant was rs2235370 at 1q32.2 in the intron of *TRAF3IP3* (*p*-value = 1.33 × 10^−8^). When we adjusted for the 8q24 effect, the associations in *IRF6* became insignificant, although a genome-wide significance was still identified in *TRAFIP3*. This may indicate that the effect of 8q24 was highly correlated with *IRF6* but not with *TRAFIP3*.

We additionally found three independent associations at a suggestive significance level (*p*-value < 1.00 × 10^−6^). Conditioning on three genome-wide significant loci, rs16957821 was identified within *NTN1* at 17p13.1 (*p*-value = 1.40 × 10^−7^), which had been previously reported by CL/P and OFC (cleft lip and/or palate) GWAS and replication studies [15,16,19,22,36]. The tenth independently associated variant (rs138322543, *p*-value = 5.43 × 10^−7^) is not within a gene, but the nearest gene is *DCAF4L2,* which was previously identified by OFC GWAS [15,16,19].

From our TWAS analyses on four tissue types (whole blood, skeletal muscle, subcutaneous adipose and visceral adipose tissues), we found nine genes with transcriptome-wide significant associations (*p*-value < 2.00 × 10^−6^) (Table 4 and Figure S3). *THEM79* and *GLMP* were predicted to be significantly associated with OFC in whole blood, *DDT* in skeletal muscle and subcutaneous adipose tissues, and *DDTL, MIF, MIF-AS1, GSTT2* and USP2 in subcutaneous adipose tissue. Previously, *MIF* was reported in association with susceptibility to juvenile arthritis [43], *GSTT2* with S-phenylmercapturic acid levels in smokers [44], and *USP2* suggestively with urate levels [45]. In the visceral adipose tissue, we identified four genes that overlapped or related to the subcutaneous adipose tissue result: *MIF*, *MIF-AS1*, *DDTL*, *GSTT2*, with the addition of *GSTT2B*. More genes with suggestive evidence of associations are described in Table S2. From the gene ontology analyses, we found that *MIF* and *DDT* were enriched in terms of intramolecular oxidoreductase activity (adjusted *p*-value = 1.31 × 10^−2^) and the activity transposing carbon-carbon double bonds (adjusted *p*-value = 3.11 × 10^−3^); *GSTT2B* and *GSTT2* were enriched in terms of glutathione transferase activity (adjusted *p*-value = 7.00 × 10^−3^), as well as in terms of the alkyl or aryl group transferase activity (adjusted *p*-value = 1.59 × 10^−2^)

We also looked up previously reported candidate genes in our four tissue-specific TWAS (Table S3). We listed 34 OFC loci reported from previous GWASs, including suggestive evidence in the largest and most recent meta-analysis [22,46]. Of these 34 candidate genes, the GTEx database contained 27 genes at 25 loci (Table S1); the number of available candidate genes varied by tissues (12 in whole blood, 11 in skeletal muscle tissue, seven in subcutaneous adipose tissue and eight in visceral adipose tissue). Bonferroni significance levels were also differently set depending on the tissues, with 0.004 for whole blood and skeletal muscle tissue, 0.007 for subcutaneous adipose tissue and 0.006 for visceral adipose tissue. *RHPN2* at 19q13.11 was identified as being Bonferroni-significant from skeletal muscle and subcutaneous adipose tissue transcriptome association tests (*p*-value = 1.90 × 10^−3^ and 1.45 × 10^−3^). In both tissues, the upregulation of *RHPN2* had a beneficial effect on the risk of OFC.

## 4. Discussion

Since we performed association analyses on OFC (cleft lip and/or palate), including “cleft palate only” cases, a direct comparison to GWASs of “cleft lip with or without cleft palate case” may not be eligible. However, we hypothesize that there is a genetic variant that has influence on the shared genetics between two related phenotypes. A recent meta-analysis performed GWAS on three cleft phenotypes, including two well-studied phenotypes, CL/P and CP, and additionally a constitute phenotype, OFC (the same as “OFC” in the study) [22], where they identified a novel locus, *FOXE1*, in association with OFC but not with CL/P or CP. *FOXE1* had been reported in linkage studies and a candidate gene setting [8,9,10] but had never been identified by prior GWAS. This study showed that seven out of ten genome-wide association signals from CL/P and CP studies could be captured with the OFC study. Three out of the ten genome-wide significant signals were not recaptured by the OFC study, and two of these (3q28 and 15q24) were from the whole population analysis, while the other one (17q23.2) was from the European analysis; however, only the last one was replicated by a multiethnic GWAS [19].

A mixed-model analysis can not only adjust for population structures, but it can also adjust for sample relatedness, making the approach especially useful for multiethnic family studies. To account for the population structure, the previous CCDG GWAS study [19] estimated the principal components for the pooled ancestry based on the subset of subjects and SNPs. On the other hand, the EMMAX analysis accounts for the population structures, including those of excluded ancestries, by building up genetic relationships between all pairs of subjects based on all available SNPs. This additional information on the genetic covariance between subjects and the population structure enabled our study to identify a comparable set of loci associated with OFC. At the genome-wide significant level (*p* = 5.00 × 10^−8^), the CCDG GWAS [19] identified seven loci in the pooled population, i.e., 19q13 near *RHPN2*, *PAX7* at 1p36, *ARHGAP29* at 1p22, *IRF6* at 1q32, 8q24 and *NTN1* at 17p13, and a novel locus 2p24 near *FAM49A*. In this study, we identified five genes at four loci (8q24, *IRF6* and *TRAF3IP3* at 1q32, *FAM49A* at 2p24 and *NTN1* at 17p13). All four loci identified by the CCDG GWAS [19] were recapitulated, and the other three genes, 19q13 near *RHPN2*, *PAX7* at 1p36 and *ARHGAP29* at 1p22, were not. However, we suggest that *TRAF3IP3* should be considered as a novel finding, one independent from *IRF6*. Compared to the traditional linkage or trio-based methods, the linear mixed-model analysis can be beneficial to a larger sample size for variants with a lower penetrance. At the suggestive significance level (*p*-value < 1.00 × 10^−6^), we identified several novel genes, *DIEXF,* another gene at 1q32.2 (*p*-value = 5.51 × 10^−8^), *DCAF4L2* at 8q21.3 (*p*-value = 3.64 × 10^−7^), and a novel locus *GMDS* at 6p25.3 (*p*-value = 6.70 × 10^−7^).

We observed that many association signals showed different allele frequency distributions between subpopulations (Table S4), which supports the idea that we need to pay extensive attention to controlling the population structure in genetic analyses. Recently, meta-analyses of multiple races showed that a set of genome-wide significant loci considerably differed by racial groups [22]. Furthermore, a GWAS in a Chinese population suggested that the roles of SNPs at the well-known 1q32.2 locus were different in the Chinese haplotype structure [17]. In this study, the most significant SNP was at the well-known 8q24 locus, and allele frequencies for this SNP (rs72728755) varied in subpopulations; the alternate allele (A) is rare in Asians and Native Americans but is quite prevalent in other populations. Allele frequencies of the top SNP at the 2p24.2 locus show that the ‘G’ allele is minor only in Caucasians but major in all other populations. In a previous CCDG GWAS [19], stratified analyses were performed on Asians and Caucasians, but this locus was not reported, which means that the 2p24.2 signal was detectable by the addition of other population samples in our mixed-model analysis. At the locus of 1q32.2, the alternate allele does not exist in Africans and is uncommon in Caucasians, while it is common in Asian, Native American and other mixed populations. The *TRAF3IP3* gene was also not previously identified from the previous CCDG analysis, but it is likely that adding other populations improved the statistical power to detect this locus.

While our initial GWAS results were consistent with previous findings [46], some signals were not independent of each other. Conditioning on 8q24, the effect of 1q32.2 diminished and strengthened another signal at 2p24.2 (*FAM49A*) which had been less significant than that from 1q32.2. This could indicate that 8q24 is strongly associated with 1q32.2 and that the effect from these loci masks the effect from 2p24.2. Interestingly, the effect of one of the most well-established loci in the OFC association, *IRF6*, was removed after adjusting for the 8q24 effect, but the effect of a marker in *TRAF3IP3* at the same locus (1q32.2) survived. *TRAF3IP3* is a gene encoding the TRAF3-interacting protein 3, and *TRAF3* encodes a TNF receptor-associated factor 3. Although *TRAF3IP3* has never been identified by previous linkage and GWA studies, the gene is proximate to *IRF6* at the 1q32.2 locus, suggesting that *TRAF3IP3* may play a regulatory role for *IRF6*. From our analysis, this *TRAF3IP3* gene was associated with a lower risk of cleft (β = −0.0518). Additionally, after adjusting the effects of 8q24, 2p24.2 and 1q32.2, one of the genome-wide significant loci from the initial analysis, 17p13.1 (*NTN1*), became insignificant. This can also suggest that the 17p13.1 locus is closely associated with the three loci adjusted in the step, especially with 8q24 and 1q32.2, which had been reported together with 17p13.11 by Leslie et al. [19].

We identified significant associations in the predicted expression TWAS, and our result may add novel evidence to OFC etiology. Two human h-class isoenzymes, *GSTT1* and *GSTT2,* are both located at 22q11.2 and are separated by approximately 50 kb. Previously, copy-number variations (CNVs) at 22q11.2 were reported in association with a low birth weight and preterm delivery outcomes [47]. An increased risk of cleft lip with or without a cleft palate has been associated with maternal smoking in children with null polymorphisms in *GSTT1*, implicating that it may modify the effect of maternal smoking by involving the removal of toxic compounds generated from smoking [48,49,50]. The expression level for *GSTT1* was not included in our TWAS, but the isoenzyme coding gene *GSTT2* was identified with a significance. The messenger RNA expression of *GSTT2* was reported as being associated with Charcot–Marie–Tooth disease, which is one of the most common inherited neurological disorders [51]. *GSTT1* and *GSTT2* genes are known to be involved in glutathione synthesis and drug detoxification [52,53]. Additionally, a GWAS of liver enzymes reported that a SNP (rs2739330) associated with a serum γ–glutamyl transferase enzyme was an eQTL for *DDT*, *DDTL*, *GSTT1* and *MIF* [54], suggesting a shared genetic control over our TWAS genes. By including another closely located TWAS significant gene, *GSTT2B,* our TWAS result may imply glutathione synthesis and a drug detoxification-related OFC etiology.

Our study exhibits several limitations. OFC prevalence varies among populations, and some genes identified from previous GWASs showed distinct patterns by population in our analyses (e.g., the desert region in 8q24) [13]. This might indicate a differential transcriptomic variation of OFC-related genes in populations. Since the GTEx database has mainly developed expression datasets for Caucasians, this study also included subjects with Caucasian ancestry only. Thus, findings from our TWAS analysis would not apply to races other than Caucasians. Furthermore, due to a lack of tissues directly related to orofacial clefts, this study carefully selected four tissues that were relatively more relevant to our biological understandings. In addition to whole blood and skeletal muscle tissue, we have taken subcutaneous and visceral tissues into account. In the fusion process developmental theory, insufficient penetration of the middle layer of the embryo (“mesoderm”) causes cleft [55,56]. A study that tracked the fate of major mesodermal subcompartments in mice demonstrated that visceral and subcutaneous adipocytes were segregated from posterior lateral plate mesoderm by late embryogenesis [5]. Visceral and subcutaneous adipose tissues were used as surrogates for the mesodermal lineage; however, studies with eQTL from orofacial tissues will provide more fundamental evidence. Last, not all of our candidate genes were included in the GTEx eQTL data. More findings on eQTLs associated with those candidate genes will help us to better understand OFC etiology. It is likely that candidate genes of interest did not show a significant association with expression levels in the GTEx database, so no eQTLs were identified. However, the GTEx project is ongoing, and more samples from diverse populations may add eQTLs associated with the candidate genes. For example, a COGENE (Craniofacial and Oral Gene Expression Network) project reported an elevated expression profile for *GSTT1* in craniofacial tissues during embryonic development, but *GSTT1* was not included in the trained expression dataset. Instead, genome-wide significant signals were identified from *GSTT2* and *GSTT2B* in this study.

## 5. Conclusions

Using a mixed-model association analysis, we confirmed previous findings for OFC as well as independence among risk loci. We have identified that unlike *IRF6,* the genome-wide significant association of *TRAF3IP3* is independent from the association signal at 8q24. Additionally, TWAS using predicted expression levels identified novel genes associated with OFC that implicate the etiology related to glutathione synthesis and drug detoxification. In conclusion, in spite of several limitations, we added meaningful evidence to the OFC etiology using novel statistical strategies.

## Figures and Tables

**Table 1:** Demographic Information.

Characteristics	Categories	Control (*n* = 7839)	Case (*n* = 2703)
Cleft Type	Cleft Lip only	-	620
	Cleft Lip and Palate	-	1771
	Cleft Palate only	-	312
Sex	Male	3397 (43.3%)	1581 (58.50%)
	Female	4442 (56.7%)	1122 (41.50%)
Age		28.66 (±16.95)	12.06 (±13.03)
Race	African	219 (2.79%)	160 (5.92%)
	Asian	1433 (18.28%)	584 (21.60%)
	Caucasian	3563 (45.45%)	1068 (39.51%)
	Caucasian and African	33 (0.42%)	11 (0.41%)
	Caucasian and Asian	2 (0.026%)	2 (0.074%)
	Caucasian and Native American	768 (9.80%)	171 (6.33%)
	Caucasian, African and Native American	1617 (20.63%)	665 (24.60%)
	Native American	152 (1.94%)	37 (1.37%)
	Unspecified	52 (0.66%)	5 (0.18%)

**Table 2:** Genome-wide significant loci from the EMMAX analysis and their significance in the original multiethnic analysis.

Locus	EMMAX Analysis							The Multiethnic Meta-Analysis [19]			
	Sentinel SNP	Chr:Pos	Ref/Alt	MAF	β (S.E.)	*p*-Value	Gene	Sentinel SNP	Meta OR ^†^	*p*-Value	r^2 ‡^
8q24	rs72728755	8:129990382	T/A	0.16	0.08 (0.008)	1.94 × 10^−22^	Gene desert	rs17242358	2.00 [1.78–2.26]	5.63 × 10^−30^	0.97
1q32.2	rs2235370	1:209946027	C/A	0.13	−0.05 (0.009)	5.15 × 10^−9^	*TRAF3IP3*	rs11119345	1.81 [1.57–2.07]	2.52 × 10^−17^	0.84
	rs2235373	1:209963803	G/A	0.26	−0.04 (0.007)	2.12 × 10^−8^	*IRF6*				0.30
2p24.2	rs7552	2:16733928	A/G	0.50	0.04 (0.006)	3.40 × 10^−8^	*FAM49A*	rs7552	1.28 [1.17–1.40]	4.22 × 10^−8^	1.00
17p13.1	rs11273201	17:8930219	AACCCAAAACCCAC/A	0.27	−0.04 (0.007)	3.58 × 10^−8^	*NTN1*	rs11273201	1.44 [1.29–1.59]	7.84 × 10^−12^	1.00

Chr = chromosome; Pos = position in base pairs (hg19); Ref = reference allele; Alt = alternate allele; MAF = minor allele frequency, S.E. = standard error of β. ^†^ Odds ratio from the meta-analysis of the case-parent trio analysis and case-control analysis. ^‡^ r^2^ LD statistics against all populations of 1000 Reference Genome.

**Table 3:** Top association from each step of the EMMAX conditional association analysis.

No.	SNP ID	Chr:Pos	Ref/Alt	MAF	β (S.E.)	*p*-Value	Locus/Gene
1	rs72728755	8:129990382	T/A	0.16	0.08 (0.008)	1.94 × 10^−22^	8q24
2	rs7552	2:16733928	A/G	0.50	0.03 (0.006)	2.67 × 10^−8^	*FAM49A*
3	rs2235370	1:209946027	C/A	0.13	−0.05 (0.009)	1.33 × 10^−8^	*TRAF3IP3*
4	rs16957821	17:8948104	C/G	0.24	0.04 (0.007)	1.40 × 10^−7^	*NTN1*
5	rs12600562	17:44977040	G/T	0.47	−0.03 (0.006)	7.06 × 10^−7^	*LRRC37A2*
6	rs12543318	8:88868340	C.A	0.45	−0.03 (0.006)	5.43 × 10^−7^	8q21
7	rs3845903	3:66517670	C/T	0.03	−0.10 (0.020)	2.79 × 10^−6^	*LRIG1*
8	rs10778143	12:102094643	A/C	0.33	0.03 (0.007)	1.76 × 10^−6^	*CHPT1*
9	rs6929507	6:1632072	T/C	0.01	0.13 (0.027)	2.14 × 10^−6^	*GMDS*
10	rs138322543	9:100641771	CCACCA/C	0.27	0.03 (0.007)	4.82 × 10^−6^	*TRMO*

Abbreviations: No. = The sequential order of the conditional analysis; Chr = chromosome; Pos = position in base pairs; Ref = reference allele; Alt = alternate allele; MAF = minor allele frequency.

**Table 4:** Genes identified from the association analysis of the predicted expression levels.

Gene	Ensembl ID	GRCh37 Coordinate	β (S.E.)	z	*p*-Value
**Whole blood**					
*THEM79*	ENSG00000163472	1: 156,252,726–156,262,976	2.71 (0.56)	4.87	1.13 × 10^−6^
*GLMP*	ENSG00000198715	1: 156,259,880–156,265,480	1.57 (0.33)	4.83	1.37 × 10^−6^
**Skeletal muscle**					
*DDT*	ENSG00000099977	22: 24,313,554–24,322,660	1.12 (0.21)	5.25	1.52 × 10^−7^
**Adipose (Subcutaneous)**					
*MIF*	ENSG00000240972	22: 24,236,191–24,237,414	1.79 (0.28)	6.39	1.62 × 10^−10^
*MIF-AS1*	ENSG00000218537	22: 24,236,613–24,241,117	1.39 (0.24)	5.87	4.25 × 10^−9^
*DDT*	ENSG00000099977	22: 24,313,554–24,322,660	2.94 (0.54)	5.43	5.66 × 10^−8^
*GSTT2*	ENSG00000099984	22: 24,322,249–24,326,106	−1.42 (0.28)	−5.16	2.46 × 10^−7^
*USP2*	ENSG00000036672	11: 119,225,925–119,252,436	−1.75 (0.38)	−4.57	4.93 × 10^−6^
*DDTL*	ENSG00000099974	22: 24,309,089–24,314,721	−1.46 (0.32)	−4.54	5.68 × 10^−6^
**Adipose (Visceral)**					
*MIF*	ENSG00000240972	22: 24,236,191–24,237,414	1.66 (0.26)	6.52	7.09 × 10^−11^
*DDTL*	ENSG00000099974	22: 24,309,089–24,314,721	−2.60 (0.45)	−5.81	6.37 × 10^−9^
*MIF-AS1*	ENSG00000218537	22: 24,236,613–24,241,117	1.10 (0.19)	5.78	7.31 × 10^−9^
*GSTT2*	ENSG00000099984	22: 24,322,249–24,326,106	−1.05 (0.19)	−5.53	3.27 × 10^−8^
*GSTT2B*	ENSG00000133433	22: 24,299,601–24,303,373	−1.36 (0.28)	−4.94	7.92 × 10^−7^

## Data Availability

Center for Craniofacial and Dental Genetics (CCDG) data on dbGaP (accession: phs000774.v2.p1).

## Supplementary Materials

The following are available online at https://www.mdpi.com/2073-4425/11/11/1280/s1, Figure S1: Quantile-quantile (QQ) plot of OFC GWAS using EMMAX model, Figure S2: QQ Plots and Manhattan Plots from Nine Sequential Conditional GWASs, Figure S3: QQ Plots of OFC TWASs Using Predicted Expression Levels in Four Tissues. Table S1: Candidate Genes in the GTEx database, Table S2: Results with suggestive evidence of association from the gene-based association study, Table S3: Candidate Gene Study Using Predicted Expression Levels, Table S4: Alternate Allele Frequencies of the Most Significant Markers from a Series of Conditional GWAS in Sub-populations.

## References

[1] Stanier P., Moore G.E. (2004). Genetics of cleft lip and palate: Syndromic genes contribute to the incidence of non-syndromic clefts. Hum. Mol. Genet..

[2] Rahimov F., Jugessur A., Murray J.C. (2011). Genetics of nonsyndromic orofacial clefts. Cleft Palate-Craniofacial J..

[3] Berk N.W., Marazita L.M. (2002). Chapter 36. Costs of cleft lip and polate: Personal and societal implications. Cleft Lip and Palate: From Origin to Treatment.

[4] Grosen D., Chevrier C., Skytthe A., Bille C., Mølsted K., Sivertsen Å., Murray J.C., Christensen K. (2009). A cohort study of recurrence patterns among more than 54,000 relatives of oral cleft cases in Denmark: Support for the multifactorial threshold model of inheritance. J. Med. Genet..

[5] Sivertsen Å., Wilcox A.J., Skjaerven R., Vindenes H.A., Åbyholm F., Harville E., Lie R.T. (2008). Familial risk of oral clefts by morphological type and severity: Population based cohort study of first degree relatives. BMJ.

[6] Grosen D., Bille C., Petersen I., Skytthe A., Hjelmborg J.V.B., Pedersen J.K., Murray J.C., Christensen K. (2011). Risk of Oral Clefts in Twins. Epidemiology.

[7] Marazita M. (2002). Chapter 18. Segregation Analyses. Cleft Lip and Palate: From Origin to Treatment.

[8] Marazita M.L., Murray J.C., Lidral A.C., Arcos-Burgos M., Cooper M.E., Goldstein T., Maher B.S., Daack-Hirsch S., Schultz R., Mansilla M.A. (2004). Meta-Analysis of 13 Genome Scans Reveals Multiple Cleft Lip/Palate Genes with Novel Loci on 9q21 and 2q32-35. Am. J. Hum. Genet..

[9] Marazita M.L., Lidral A.C., Murray J.C., Field L., Maher B.S., McHenry T.G., Cooper M.E., Govil M., Daack-Hirsch S., Riley B. (2009). Genome Scan, Fine-Mapping, and Candidate Gene Analysis of Non-Syndromic Cleft Lip with or without Cleft Palate Reveals Phenotype-Specific Differences in Linkage and Association Results. Hum. Hered..

[10] Ludwig K.U., Böhmer A.C., Rubini M., Mossey P.A., Herms S., Nowak S., Reutter H., Alblas M.A., Lippke B., Barth S. (2014). Strong Association of Variants around FOXE1 and Orofacial Clefting. J. Dent. Res..

[11] Birnbaum S., Ludwig K.U., Reutter H.M., Herns S., Steffens M., Rubini M., Baluardo C., Ferrian M., De Assis N.A., Alblas M.A. (2009). Key susceptibility locus for nonsyndromic cleft lip with or without cleft palate on chromosome 8q24. Nat. Genet..

[12] Grant S.F., Wang K., Zhang H., Glaberson W., Annaiah K., Kim C.E., Bradfield J.P., Glessner J.T., Thomas K.A., Garris M. (2009). A Genome-Wide Association Study Identifies a Locus for Nonsyndromic Cleft Lip with or without Cleft Palate on 8q24. J. Pediatr..

[13] Beaty T.H., Murray J.C., Marazita M.L., Munger R.G., Ruczinski I., Hetmanski J.B., Liang K.Y., Wu T., Murray T., Fallin M.D. (2010). A genome-wide association study of cleft lip with and without cleft palate identifies risk variants near MAFB and ABCA4. Nat. Genet..

[14] Mangold E., Ludwig K.U., Birnbaum S., Baluardo C., Ferrian M., Herms S., Reutter H.M., De Assis N.A., Al Chawa T., Mattheisen M. (2009). Genome-wide association study identifies two susceptibility loci for nonsyndromic cleft lip with or without cleft palate. Nat. Genet..

[15] Ludwig K.U., Mangold E., Herms S., Nowak S., Reutter H., Paul A., Becker J., Herberz R., AlChawa T., Nasser E. (2012). Genome-wide meta-analyses of nonsyndromic cleft lip with or without cleft palate identify six new risk loci. Nat. Genet..

[16] Beaty T.H., Taub M.A., Scott A.F., Murray J.C., Marazita M.L., Schwender H., Parker M., Hetmanski J.B., Balakrishnan P., Mansilla M.A. (2013). Confirming genes influencing risk to cleft lip with/without cleft palate in a case-parent trio study. Hum. Genet..

[17] Sun Y., Huang Y., Yin A., Pan Y., Wang Y., Wang C., Du Y., Wang M., Lan F., Hu Z. (2015). Genome-wide association study identifies a new susceptibility locus for cleft lip with or without a cleft palate. Nat. Commun..

[18] Leslie E.J., Liu H., Carlson J.C., Shaffer J.R., Feingold E., Wehby G., Laurie C.A., Jain D., Laurie C.C., Doheny K.F. (2016). A Genome-wide Association Study of Nonsyndromic Cleft Palate Identifies an Etiologic Missense Variant in GRHL3. Am. J. Hum. Genet..

[19] Leslie E.J., Carlson J.C., Shaffer J.R., Feingold E., Wehby G., Laurie C.A., Jain D., Laurie C.C., Doheny K.F., McHenry T. (2016). A multi-ethnic genome-wide association study identifies novel loci for non-syndromic cleft lip with or without cleft palate on 2p24.2, 17q23 and 19q13. Hum. Mol. Genet..

[20] Mangold E., Böhmer A.C., Ishorst N., Hoebel A.-K., Gültepe P., Schuenke H., Klamt J., Hofmann A., Gölz L., Raff R. (2016). Sequencing the GRHL3 Coding Region Reveals Rare Truncating Mutations and a Common Susceptibility Variant for Nonsyndromic Cleft Palate. Am. J. Hum. Genet..

[21] Leslie E.J., Carlson J.C., Shaffer J.R., Buxó C.J., Castilla E.E., Christensen K., Deleyiannis F.W.B., Field L.L., Hecht J.T., Moreno L. (2017). Association studies of low-frequency coding variants in nonsyndromic cleft lip with or without cleft palate. Am. J. Med Genet. Part A.

[22] Leslie E.J., Carlson J.C., Shaffer J.R., Butali A., Buxó C.J., Castilla E.E., Christensen K., Deleyiannis F.W.B., Field L.L., Hecht J.T. (2017). Genome-wide meta-analyses of nonsyndromic orofacial clefts identify novel associations between FOXE1 and all orofacial clefts, and TP63 and cleft lip with or without cleft palate. Hum. Genet..

[23] Yu Y., Zuo X., He M., Gao J., Fu Y., Qin C., Meng L., Wang W., Song Y., Cheng Y. (2017). Genome-wide analyses of non-syndromic cleft lip with palate identify 14 novel loci and genetic heterogeneity. Nat. Commun..

[24] Eshete M., Liu H., Li M., Adeyemo W., Gowans L., Mossey P., Busch T., Deressa W., Donkor P., Olaitan P. (2017). Loss-of-Function GRHL3 Variants Detected in African Patients with Isolated Cleft Palate. J. Dent. Res..

[25] Beaty T.H., Ruczinski I., Murray J.C., Marazita M.L., Munger R.G., Hetmanski J.B., Murray T., Redett R.J., Fallin M.D., Liang K.Y. (2011). Evidence for gene-environment interaction in a genome wide study of nonsyndromic cleft palate. Genet. Epidemiol..

[26] Haaland Ø.A., Lie R.T., Romanowska J., Gjerdevik M., Gjessing H.K., Jugessur A. (2018). A Genome-Wide Search for Gene-Environment Effects in Isolated Cleft Lip with or without Cleft Palate Triads Points to an Interaction between Maternal Periconceptional Vitamin Use and Variants in ESRRG. Front. Genet..

[27] Curà F., Palmieri A., Girardi A., Carinci F., Morselli P.G., Nouri N., Pezzetti F., Scapoli L., Martinelli M. (2018). Possible effect of SNAIL family transcriptional repressor 1 polymorphisms in non-syndromic cleft lip with or without cleft palate. Clin. Oral Investig..

[28] Estandia-Ortega B., Velázquez-Aragón J.A., Alcántara-Ortigoza M.A., Reyna-Fabián M.E., Villagómez-Martínez S., Angel A.G.-D. (2014). 5,10-Methylenetetrahydrofolate reductase single nucleotide polymorphisms and gene-environment interaction analysis in non-syndromic cleft lip/palate. Eur. J. Oral Sci..

[29] Wu T., Fallin M.D., Shi M., Ruczinski I., Liang K.Y., Hetmanski J.B., Wang H., Ingersoll R.G., Huang S., Ye X. (2012). Evidence of gene-environment interaction for the RUNX2 gene and environmental tobacco smoke in controlling the risk of cleft lip with/without cleft palate. Birth Defects Res. Part A Clin. Mol. Teratol..

[30] Niktabar S.M., Aarafi H., Dastgheib S.A., NooriShadkam M., Mirjalili S.R., Lookzadeh M.H., Akbarian-Bafghi M.J., Morovati-Sharifabad M., Neamatzadeh H. (2019). Association of MTHFR 1298A > C Polymorphism with Susceptibility to Non-Syndromic Cleft Lip with or without Palate: A Case-Control Study and Meta-Analysis. Fetal Pediatr. Pathol..

[31] Li W., Wang M., Zhou R., Wang S., Zheng H., Liu D., Zhou Z., Zhu H., Wu T., Beaty T.H. (2019). Exploring the interaction between FGF Genes and T-box genes among chinese nonsyndromic cleft lip with or without cleft palate case-parent trios. Environ. Mol. Mutagen..

[32] Jugessur A., Rahimov F., Lie R.T., Wilcox A.J., Gjessing H.K., Nilsen R.M., Nguyen T.T., Murray J.C. (2008). Genetic variants inIRF6and the risk of facial clefts: Single-marker and haplotype-based analyses in a population-based case-control study of facial clefts in Norway. Genet. Epidemiol..

[33] Rahimov F., Program N.C.S., Marazita M.L., Visel A., Cooper M.E., Hitchler M.J., Rubini M., Domann F.E., Govil M., Christensen K. (2008). Disruption of an AP-2α binding site in an IRF6 enhancer is associated with cleft lip. Nat. Genet..

[34] Ingraham C.R., Kinoshita A., Kondo S., Yang B., Sajan S., Trout K.J., Malik M.I., Dunnwald M., Goudy S.L., Lovett M. (2006). Abnormal skin, limb and craniofacial morphogenesis in mice deficient for interferon regulatory factor 6 (Irf6). Nat. Genet..

[35] Thompson J., Mendoza F., Tan E., Bertol J.W., Gaggar A.S., Jun G., Biguetti C., Fakhouri W.D. (2019). A cleft lip and palate gene, Irf6, is involved in osteoblast differentiation of craniofacial bone. Dev. Dyn..

[36] Leslie E.J., Taub M.A., Liu H., Steinberg K.M., Koboldt D.C., Zhang Q., Carlson J.C., Hetmanski J.B., Wang H., Larson D.E. (2015). Identification of Functional Variants for Cleft Lip with or without Cleft Palate in or near PAX7, FGFR2, and NOG by Targeted Sequencing of GWAS Loci. Am. J. Hum. Genet..

[37] Kang H.M., Sul J.H., Service S.K., Zaitlen N.A., Kong S.-Y., Freimer N.B., Sabatti C., Eskin E. (2010). Variance component model to account for sample structure in genome-wide association studies. Nat. Genet..

[38] Gibson G. (2015). GTEx detects genetic effects. Science.

[39] Gamazon E.R., Wheeler H.E., Shah K.P., Mozaffari S.V., Aquino-Michaels K., Carroll R.J., Eyler A.E., Denny J.C., Nicolae D.L., Cox N.J. (2015). A gene-based association method for mapping traits using reference transcriptome data. Nat. Genet..

[40] Liberzon A., Birger C., Thorvaldsdóttir H., Ghandi M., Mesirov J.P., Tamayo P. (2015). The Molecular Signatures Database Hallmark Gene Set Collection. Cell Syst..

[41] Watanabe K., Taskesen E., Van Bochoven A., Posthuma D. (2017). Functional mapping and annotation of genetic associations with FUMA. Nat. Commun..

[42] Ehuppi K., Pitt J.J., Wahlberg B.M., Caplen N.J. (2012). The 8q24 Gene Desert: An Oasis of Non-Coding Transcriptional Activity. Front. Genet..

[43] Glass D.N., Giannini E.H. (1999). Juvenile rheumatoid arthritis as a complex genetic trait. Arthritis Rheum..

[44] Haiman C.A., Patel Y.M., Stram D.O., Carmella S.G., Chen M., Wilkens L.R., Le Marchand L., Hecht S.S. (2016). Benzene Uptake and Glutathione S-transferase T1 Status as Determinants of S-Phenylmercapturic Acid in Cigarette Smokers in the Multiethnic Cohort. PLoS ONE.

[45] Koettgen A., Albrecht E., Teumer A., Vitart V., Krumsiek J., Hundertmark C., Pistis G., Ruggiero D., O’Seaghdha C.M., Haller T. (2012). Genome-wide association analyses identify 18 new loci associated with serum urate concentrations. Nat. Genet..

[46] Beaty T.H., Marazita M.L., Leslie E.J. (2016). Genetic factors influencing risk to orofacial clefts: Today’s challenges and tomorrow’s opportunities. F1000Research.

[47] Zheng X., Feingold E., Ryckman K.K., Shaffer J.R., Boyd H.A., Feenstra B., Melbye M., Marazita M.L., Murray J.C., Cuenco K.T. (2013). Association of maternal CNVs in GSTT1/GSTT2 with smoking, preterm delivery, and low birth weight. Front. Genet..

[48] Lammer E.J., Shaw G.M., Iovannisci D.M., Finnell R.H. (2005). Maternal Smoking, Genetic Variation of Glutathione S-Transferases, and Risk for Orofacial Clefts. Epidemiology.

[49] Hozyasz K.K., Mostowska A., Surowiec Z., Jagodziński P.P. (2005). Genetic polymorphisms of GSTM1 and GSTT1 in mothers of children with isolated cleft lip with or without cleft palate. Przeglad Lek..

[50] Shi M., Christensen K., Weinberg C.R., Romitti P., Bathum L., Lozada A., Morris R.W., Lovett M., Murray J.C. (2007). Orofacial Cleft Risk Is Increased with Maternal Smoking and Specific Detoxification-Gene Variants. Am. J. Hum. Genet..

[51] Kitani-Morii F., Imamura K., Kondo T., Ohara R., Enami T., Shibukawa R., Yamamoto T., Sekiguchi K., Toguchida J., Mizuno T. (2017). Analysis of neural crest cells from Charcot–Marie–Tooth disease patients demonstrates disease-relevant molecular signature. NeuroReport.

[52] Bolt H.M., Thier R. (2006). Relevance of the Deletion Polymorphisms of the Glutathione S-Transferases GSTT1 and GSTM1 in Pharmacology and Toxicology. Curr. Drug Metab..

[53] Zhang H., Forman H.J., Choi J. (2005). γ-glutamyl transpeptidase in glutathione biosynthesis. Methods Enzymol..

[54] Chambers J.C., Zhang W., Sehmi J., Li X., Wass M.N., Van Der Harst P., Holm H., Sanna S., Kavousi M., Baumeister S.E. (2011). Genome-wide association study identifies loci influencing concentrations of liver enzymes in plasma. Nat. Genet..

[55] Mishra S., Sabhlok S., Panda P.K., Khatri I. (2015). Management of Midline Facial Clefts. J. Maxillofac. Oral Surg..

[56] Sebo Z.L., Jeffery E., Holtrup B., Rodeheffer M.S. (2018). A mesodermal fate map for adipose tissue. Development.

