# Supplementary material for: Secondary Genome-Wide Association Study Using Novel Analytical Strategies Disentangle Genetic Components of Cleft Lip and/or Cleft Palate in 1q32.2
Source: Genes (Basel). 2020 Oct 29;11(11):1280. doi: 10.3390/genes11111280 (PMC7693579; doi:10.3390/genes11111280)
Supplement: Supplementary file 1 [file genes-11-01280-s001.pdf]

Table S1 Candidate Genes in the GTEx database

Gene	Locus	Phenotype	Study Type	Reference
ARHGAP29	1p22.1	CL/P	GWAS	[15, 17, 19]
GRHL3	1p36.11	CP	GWAS, GWAS replication	[14, 18]
IRF6	1q32.2	CL/P	Linkage, GWAS, GWAS replication	[8, 9, 11–15, 17, 19, 23]
TGFA	2p13	CL/P	Linkage	[9]
THADA	2p21	CL/P	GWAS meta-analysis	[16]
FAM49A	2p24	CL/P	GWAS	[17, 19]
COL8A1	3q12	CL/P	GWAS, GWAS replication, GWAS meta-analysis	[14, 19, 23]
TP63	3q27–28	CL/P	Linkage, GWAS	[9, 19]
SHROOM3	4q21.1	CL/P	GWAS meta-analysis	[19]
PIK3R1	5q13.1	CL/P	GWAS meta-analysis	[19]
NRG1	8p12	CL/P	GWAS meta-analysis	[19]
BAALC	8q22.3	CP & multivitamins	GWAS X E	[20]
SMC2	9q31.1	CP & maternal alcohol	GWAS X E	[20]
TBK1	12q14	CP & maternal smoking	GWAS X E	[20]
KRT18	12q13.13	CL/P	GWAS meta-analysis	[19]
PAX9	14q21–24	CL/P	Linkage	[8, 9]
TGFB3				
TPM1	15q22	CL/P	GWAS meta-analysis	[16]
ARID3B	15q24	CL/P	GWAS	[17, 19]
CREBBP	16p13	CL/P	GWAS	[27]
CRISPLD2	16q24	CL/P	Linkage	[9]
WNT9B	17q21.32	CP	GWAS meta-analysis	[19]
GOSR2				
TANC2	17q23.2	CL/P	GWAS	[17]
ZNF236	18q22	CP & maternal smoking	GWAS X E	[20]
RHPN2	19q13.11	CL/P	GWAS	[17]
MAFB	20q12	CL/P	GWAS	[15, 17, 19]

Table S2 Results with suggestive evidence of association from the gene-based association study

Gene	Ensembl Transcript ID	Chr; Pos	Beta (S.E.)	z	p-value
Whole Blood					
IRGM	ENSG00000237693.4	5; 150,846,523-150,900,736	-3.1560 (0.7636)	-4.1328	3.58E-05
OSBPL11	ENSG00000144909.7	3; 125,528,858-125,595,090	-2.0234 (0.4949)	-4.0887	4.34E-05
SYCE3	ENSG00000217442.3	22; 50,551,112-50,562,905	3.3258 (0.8238)	4.0371	5.41E-05
ALDH5A1	ENSG00000112294.8	6; 24,494,852-24,537,207	-1.8371 (0.4635)	-3.9633	7.39E-05
PRSS45	ENSG00000188086.8	3;46,742,092-46,744,755	2.9393 (0.7508)	3.9150	9.04E-05
Skeletal Muscle					
ATP6V1D	ENSG00000100554.7	14; 67,294,371-67,360,265	2.4795 (0.5800)	4.2750	1.91E-05
HSD17B12	ENSG00000149084.7	11; 43,680,558-43,856,617	-0.4123 (0.0991)	-4.1609	3.17E-05
MIF-AS1	ENSG00000218537.1	22; 23,894,426-23,898,930	0.7923 (0.1948)	4.0666	4.77E-05
CD151	ENSG00000177697.13	11; 832,843-839,831	-0.718 (0.1769)	-4.0581	4.95E-05
SQOR	ENSG00000137767.9	15; 45,631,148-45,691,294	-2.9416 (0.7321)	-4.0179	5.87E-05
LYSMD1	ENSG00000163155.7	1; 151,159,748-151,165,948	1.7668 (0.4436)	3.9829	6.81E-05
DHX30	ENSG00000132153.10	3; 47,802,909-47,850,195	6.5430 (1.6673)	3.9242	8.70E-05
Adipose (Subcutaneous)					
ALX4	ENSG00000052850.5	11; 44,260,444-44,310,166	-0.9276 (0.2179)	-4.2564	2.08E-05
KDM1B	ENSG00000165097.9	6; 18,155,329-18,223,853	-1.4586 (0.3619)	-4.0300	5.58E-05
SFSWAP	ENSG00000061936.5	12; 131,711,081-131,799,737	-2.0699 (0.5166)	-4.0065	6.16E-05
GSTT2B	ENSG00000133433.6	22; 23,957,414-23,961,186	-1.0478 (0.2674)	-3.9185	8.91E-05
Adipose (Visceral)					
MX2	ENSG00000183486.8	21; 41,361,943-41,409,390	-2.0284 (0.4610)	-4.4001	1.08E-05
GSTP1	ENSG00000084207.11	11; 67,583,595-67,586,656	1.8047 (0.4145)	4.3535	1.34E-05
ADAMTS13	ENSG00000160323.14	9; 33,414,358-133,459,402	-1.2160 (0.3068)	-3.9634	7.39E-05
HSD17B12	ENSG00000149084.7	11; 43,680,558-43,856,617	-0.3960 (0.1005)	-3.9410	8.11E-05
CNTNAP1	ENSG00000108797.7	17; 42,682,613-42,699,814	-1.1957 (0.3065)	-3.9012	9.57E-05

Chr: Chromosome, Pos: Position (basepair; bp)

Table S3 Candidate Gene Study Using Predicted Expression Levels

Gene	Transcript ID	Chr; Pos	Beta (SE)	z	p
**Whole Blood**					
ARID3B	ENSG00000179361	15;74,541,177-74,598,131	-1.54 (0.59)	-2.61	0.0090
CRISPLD2	ENSG00000103196	16;84,819,984-84,920,768	0.64 (0.51)	1.27	0.2033
TBK1	ENSG00000183735	12;64,451,880-64,502,108	-0.48 (0.41)	-1.16	0.2448
IRF6	ENSG00000117595	1;209,785,623-209,806,175	0.17 (0.16)	1.07	0.2858
SMC2	ENSG00000136824	9;104,094,260-104,141,417	0.44 (0.46)	0.96	0.3348
BAALC	ENSG00000164929	8;103,140,710-103,230,305	0.40 (0.43)	0.92	0.3509
TGFB3	ENSG00000119699	14;75,958,099-75,982,991	-0.27 (0.36)	-0.73	0.4658
TGFA	ENSG00000163235	2;70,447,280-70,554,193	0.27 (0.37)	0.73	0.4662
KRT18	ENSG00000111057	12;52,948,871-52,952,901	-2.99(10.41)	-0.29	0.7743
TANC2	ENSG00000170921	17;63,009,556-63,427,699	1.94 (8.53)	0.23	0.8200
NRG1	ENSG00000157168	8;31,639,386-32,767,959	-0.02 (0.22)	-0.11	0.9104
ZNF236	ENSG00000130856	18;76,822,607-76,970,727	-0.02 (0.49)	-0.05	0.9578
**Skeletal Muscle**					
RHPN2	ENSG00000131941	19; 32,978,593-33,064,888	-1.9960 (0.64)	-3.11	0.0019*
SHROOM3	ENSG00000138771	4; 76,435,100-76,783,253	0.6720 (0.27)	2.46	0.0138
PIK3R1	ENSG00000145675	5; 68,215,720-68,301,821	1.2415 (0.51)	2.45	0.0143
TBK1	ENSG00000183735	12; 64,451,880-64,502,108	-0.5811 (0.37)	-1.56	0.1193
COL8A1	ENSG00000144810	3; 99,638,475-99,799,226	0.2524 (0.25)	0.99	0.3205
TGFA	ENSG00000163235	2; 70,447,280-70,554,193	0.3384 (0.37)	0.91	0.3613
TANC2	ENSG00000170921	17; 63,009,556-63,427,699	-0.3166 (0.58)	-0.57	0.5701
ZNF236	ENSG00000130856	18; 76,822,607-76,970,727	-0.4588 (0.84)	-0.54	0.5866
GOSR2	ENSG00000108433	17; 46,923,075-46,975,524	-0.1294 (0.26)	-0.51	0.6119
SMC2	ENSG00000136824	9; 104,094,260-104,141,417	-0.4881 (1.05)	-0.46	0.6431
MAFB	ENSG00000204103	20; 40,685,848-40,689,240	0.1451 (0.65)	0.23	0.8237
**Adipose (Subcutaneous)**					
RHPN2	ENSG00000131941	19; 32,978,593-33,064,888	-0.61 (0.19)	-3.19	0.0014*
IRF6	ENSG00000117595	1; 209,785,623-209,806,175	0.34 (0.20)	1.69	0.0910
TP63	ENSG00000073282	3; 189,631,416-189,897,279	-0.36 (0.39)	-0.94	0.3497
THADA	ENSG00000115970	2; 43,230,836-43,596,046	0.32 (0.48)	0.67	0.5014
TGFB3	ENSG00000119699	14; 75,958,099-75,982,991	-0.18 (0.42)	-0.44	0.6582
TPM1	ENSG00000140416	15; 63,042,632-63,071,915	0.07 (0.22)	0.34	0.7336
KRT18	ENSG00000111057	12; 52,948,871-52,952,901	-261.49 (2.18)	3.43	NA
**Adipose (Visceral)**					
SHROOM3	ENSG00000138771	4; 76,435,100-76,783,253	-1.28 (0.58)	-2.22	0.0262
ARHGAP29	ENSG00000137962	1; 94,148,988-94,275,068	0.29 (0.22)	1.34	0.1808
ZNF236	ENSG00000130856	18; 76,822,607-76,970,727	-0.45 (0.39)	-1.15	0.2495
TPM1	ENSG00000140416	15; 63,042,632-63,071,915	-0.11 (0.41)	-0.27	0.7847
THADA	ENSG00000115970	2; 43,230,836-43,596,046	-0.14 (0.51)	-0.27	0.7885
IRF6	ENSG00000117595	1; 209,785,623-209,806,175	0.04 (0.27)	0.16	0.8708
COL8A1	ENSG00000144810	3; 99,638,475-99,799,226	-0.0064 (0.31)	-0.02	0.9836
SHROOM3	ENSG00000138771	4; 76,435,100-76,783,253	-1.28 (0.58)	-2.22	0.0262

Chr: Chromosome, Pos: Position (basepair; bp)

Table S4 Alternate Allele Frequencies of the Most Significant Markers from a Series of Conditional GWAS in Sub-populations

Step	Marker	Gene	Ref allele	Alt allele	African (n=379)	Asian (n=2,017)	Caucasian (n=4,631)	Native American (n=189)	Caucasian and Native American (n=939)	Caucasian, African and Native American (n=2,282)	Total (n=10,542)
1	*rs72728755*	8q24	T	A	0.15	0.03	0.23	0.04	0.06	0.19	0.16
2	*rs7552*	FAM49A	A	G	0.68	0.74	0.34	0.73	0.63	0.52	0.50
3	*rs2235370*	TRAF3IP3	C	A	-	0.18	0.04	0.40	0.35	0.18	0.13
4	*rs16957821*	NTN1	C	G	0.41	0.18	0.19	0.35	0.37	0.30	0.24
5	*rs12600562*	LRRC37A2	G	T	0.48	0.38	0.59	0.46	0.52	0.55	0.53
6	*rs12543318*		C	A	0.68	0.44	0.62	0.49	0.48	0.49	0.55
7	*rs3845903*	LRIG1	C	T	0.80	0.94	-	0.99	0.98	0.97	0.97
8	*rs10778143*	CHPT1	A	C	0.63	0.59	0.25	0.20	0.21	0.25	0.33
9	*rs6929507*	GMDS	T	C	0.15	0.002	-	-	0.007	0.02	0.01
10	*rs138322543*	TRMO	CCACCA	C	0.77	0.91	0.66	0.66	0.67	0.72	0.73

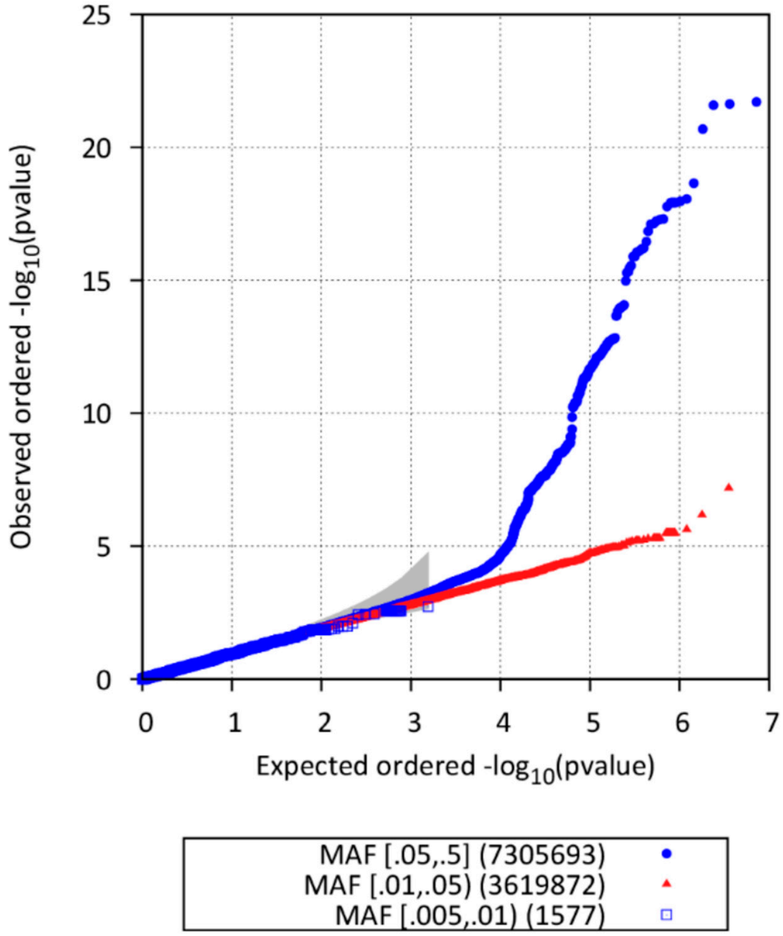

Figure S1 Quantile-quantile (QQ) plot of OFC GWAS using EMMAX model The observed (Y-axis) -log10 (p-values) are plotted against the expected (X-axis) -log10 (p-values). Common SNPs (minor allele frequency (MAF) greater than 0.05) are plotted with blue filled dots; rare SNPs (MAF smaller than 0.05 and greater than or equal to 0.01 with red filled triangles; very rare SNPs (MAF smaller than 0.01 and greater than or equal to 0.005) with blue hollow squares. Numbers of SNPs for each MAF categories are presented in the bottom box. Grey area represents a 95% confidence interval of normal distribution.

Step 1: Conditioned on 8q24

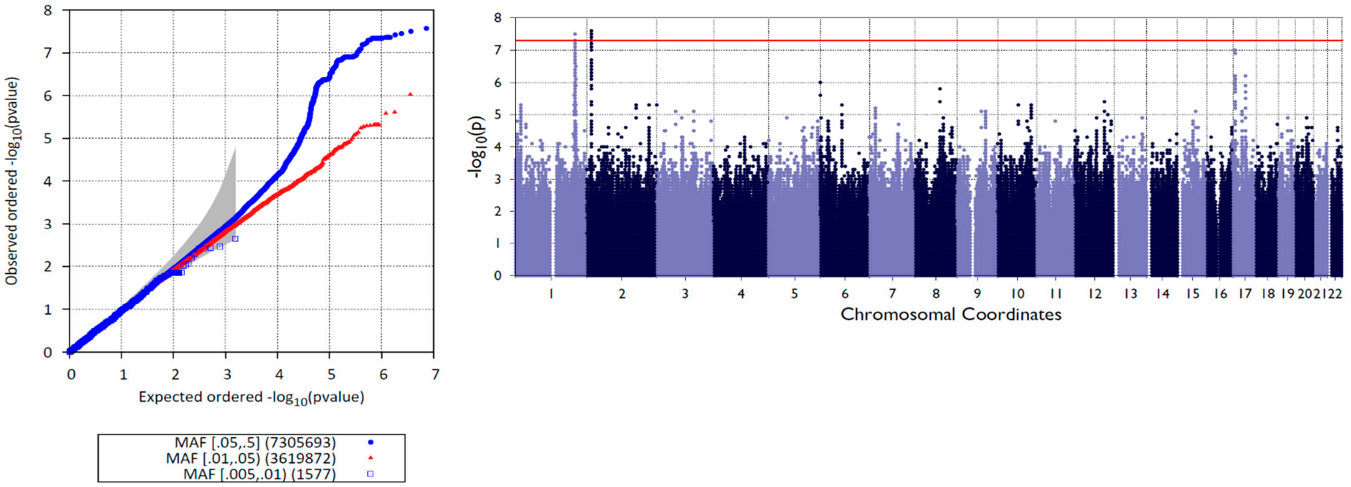

Step 2: Conditioned on 8q24 and 2p24.2

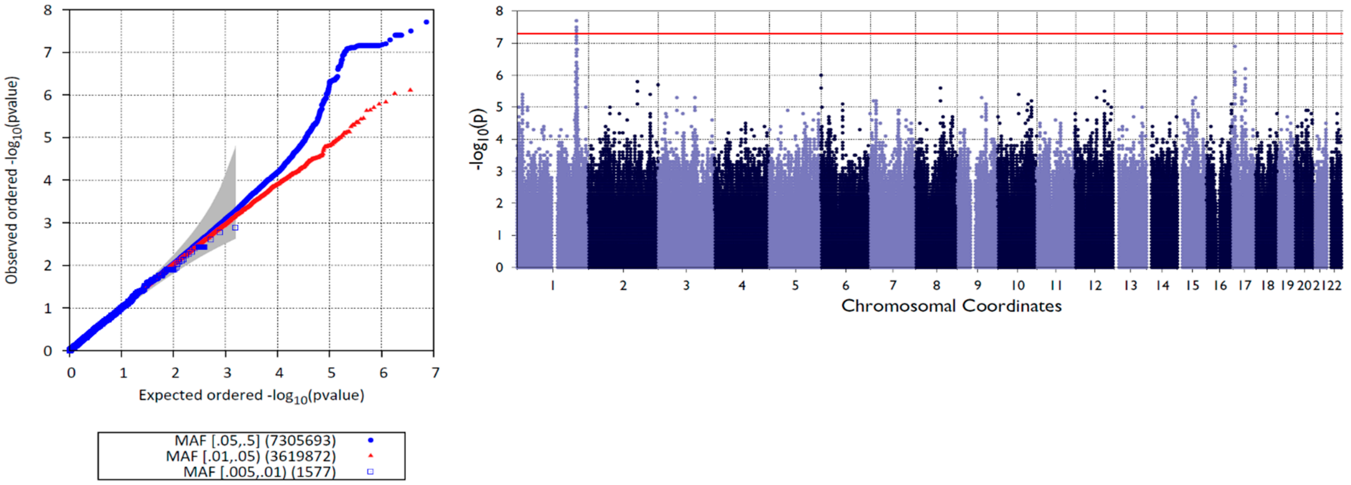

Step 3: Conditioned on 8q24, 2p24.2 and 1q32.2

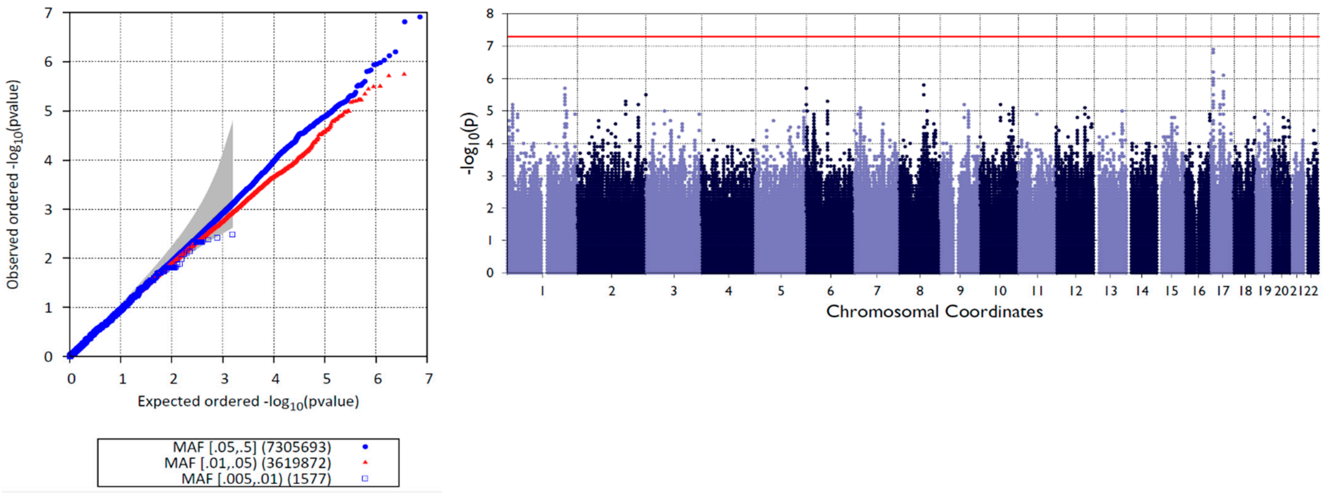

Step 4: Conditioned on 8q24, 2p24.2, 1q32.2 and 17p13.1

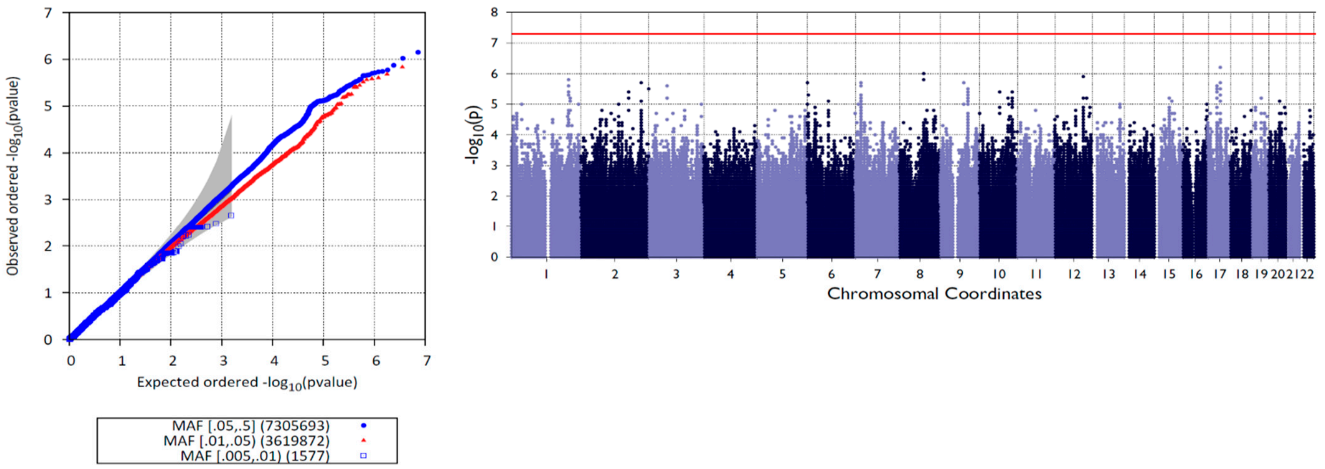

Step 5: Conditioned on 8q24, 2p24.2, 1q32.2, 17p13.1 and 17q21.31

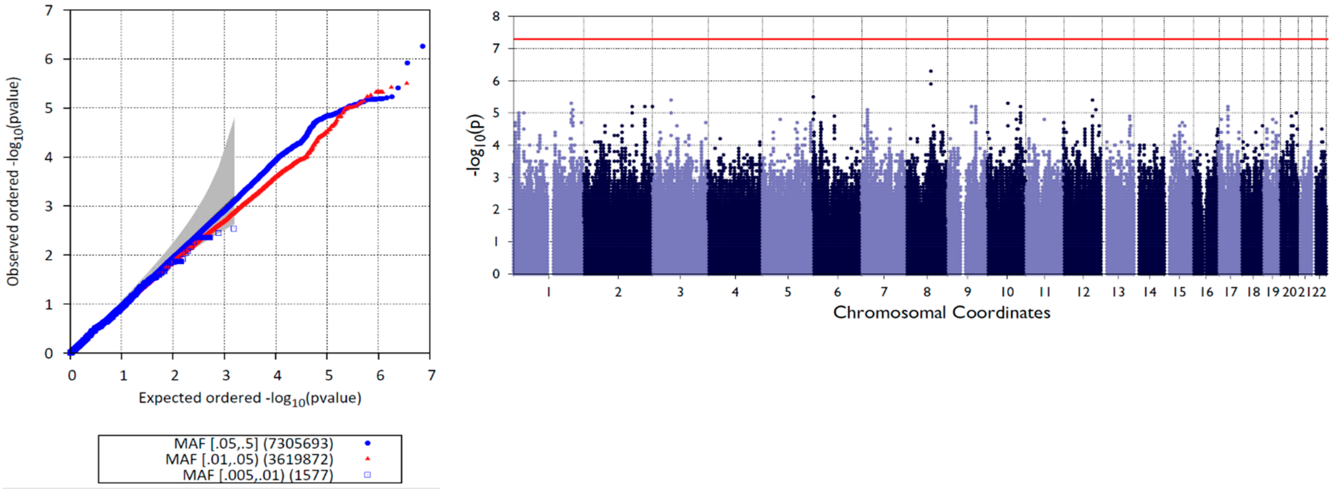

Step 6: Conditioned on 8q24, 2p24.2, 1q32.2, 17p13.1, 17q21.31 and 8q21

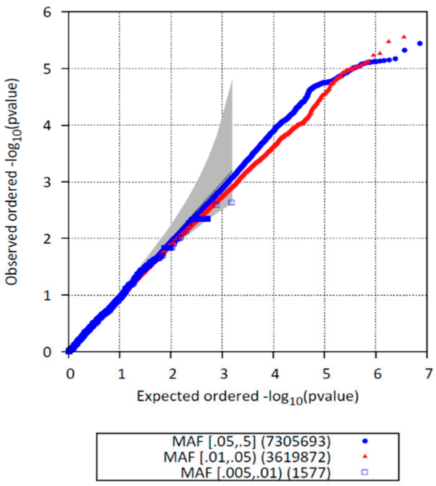

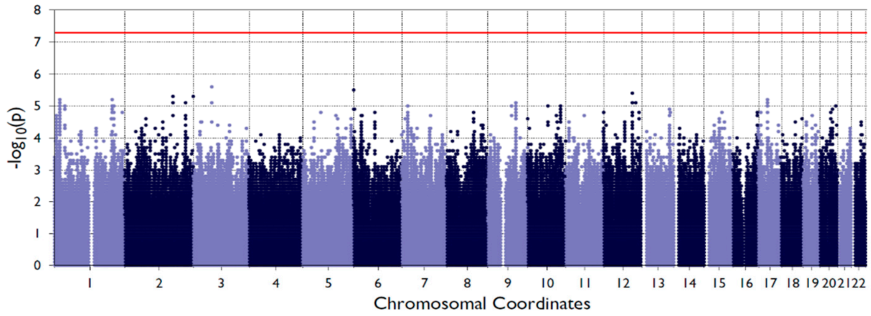

Step 7: Conditioned on 8q24, 2p24.2, 1q32.2, 17p13.1, 17q21.31, 8q21 and 3p14.1

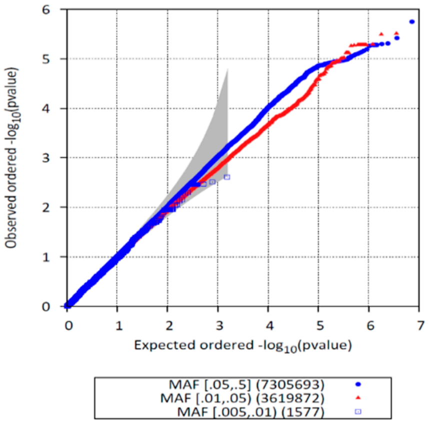

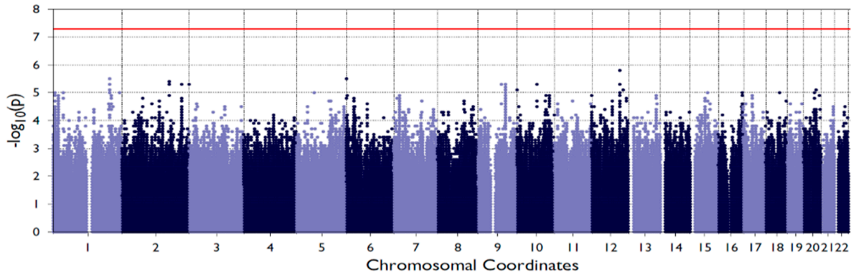

Step 8: Conditioned on 8q24, 2p24.2, 1q32.2, 17p13.1, 17q21.31, 8q21, 3p14.1 and 12q23.2

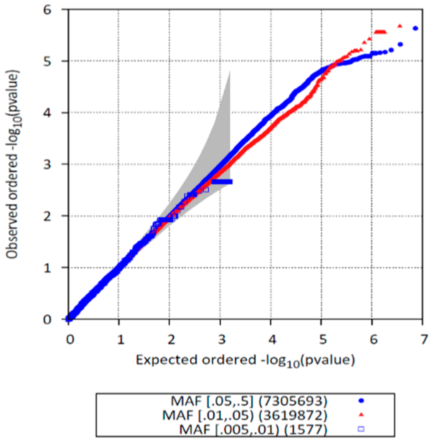

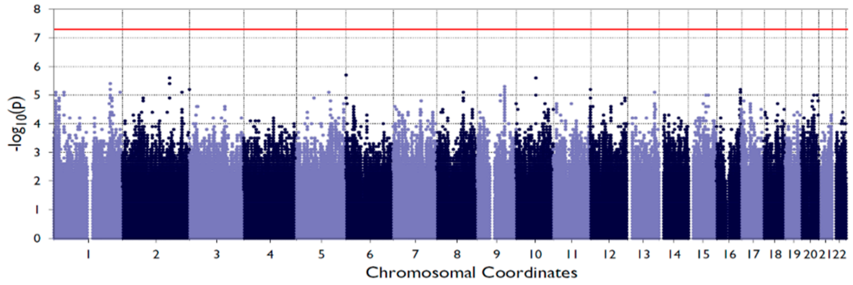

Step 9: Conditioned on 8q24, 2p24.2, 1q32.2, 17p13.1, 17q21.31, 8q21, 3p14.1, 12q23.2 and 6p25.3

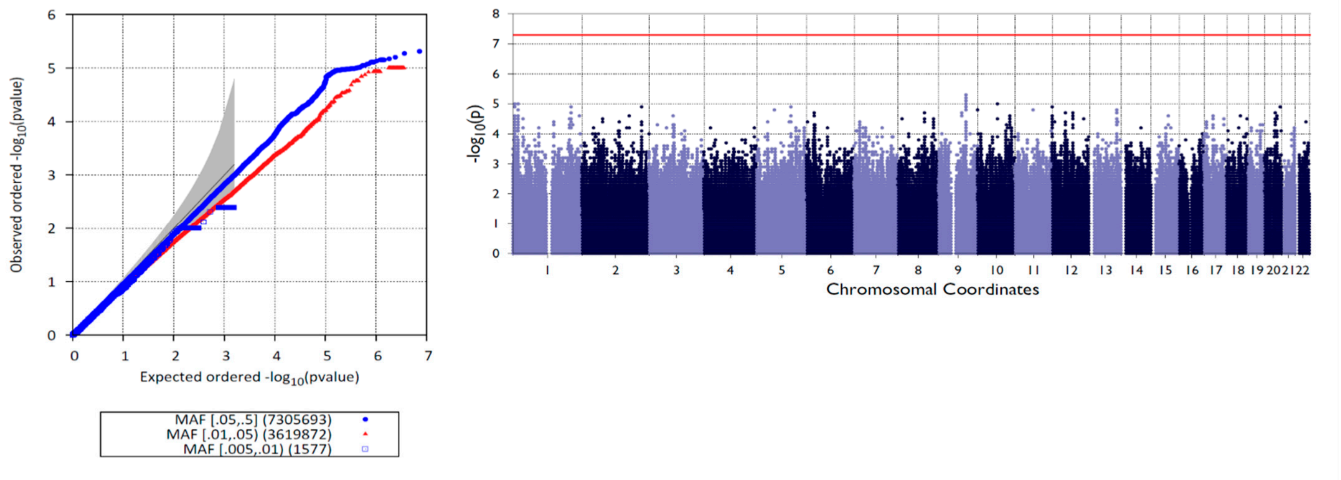

Figure S2 QQ Plots and Manhattan Plots from Nine Sequential Conditional GWASs At each step of 9 sequential GWASs, the observed (Y-axis) -log10 (p-values) are plotted against the expected (X-axis) -log10 (p-values). Manhattan plots show SNPs with -log10(p-value) on y-axis along the x-axis of chromosomal position.

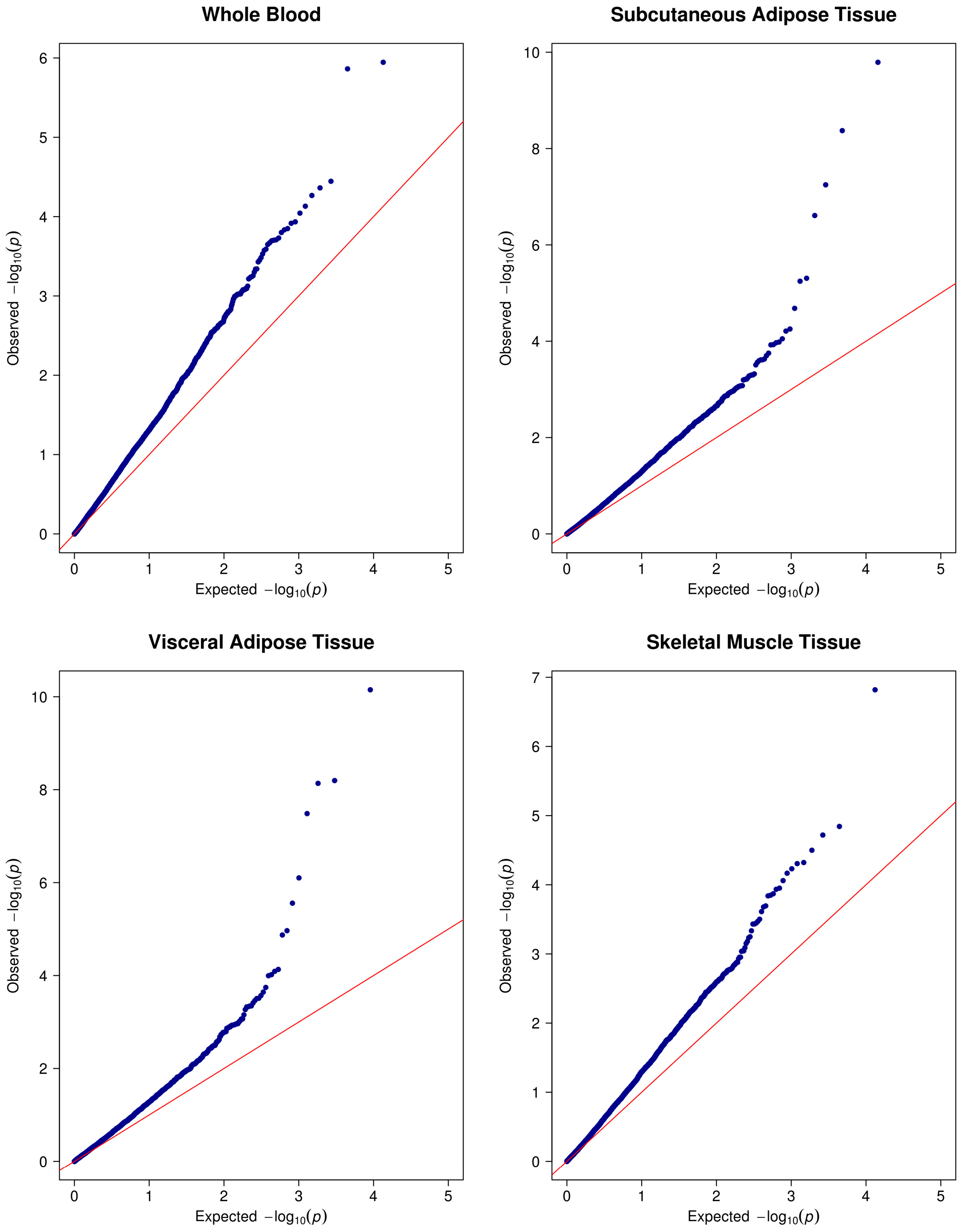

Figure S3 QQ Plots of OFC TWASs Using Predicted Expression Levels in Four Tissues The observed (Y-axis) -log10 (p-values) are plotted against the expected (X-axis) -log10 (p-values).
